# Evaluation of three fish-derived probiotic bacteria replacing antibiotics on growth, immunity, gut morphology and disease resistance in juvenile olive flounder *Paralichthys olivaceus* fed reduced fish meal diets

**DOI:** 10.3389/fnut.2025.1519140

**Published:** 2025-02-13

**Authors:** Wonsuk Choi, Mohammad Moniruzzaman, Seunghan Lee, Jinho Bae, Sungchul C. Bai, Taesun Min, Seunghyung Lee

**Affiliations:** ^1^Feeds and Foods Nutrition Research Center, Pukyong National University, Busan, Republic of Korea; ^2^Department of Animal Biotechnology, Jeju International Animal Research Center, Sustainable Agriculture Research Institute (SARI), Jeju National University, Jeju, Republic of Korea; ^3^Department of Aquaculture and Aquatic Science, Kunsan National University, Gunsan, Republic of Korea; ^4^Aquafeed Research Center, National Institute of Fisheries Science, Pohang, Republic of Korea; ^5^FAO World Fisheries University Pilot Program, Busan, Republic of Korea; ^6^Department of Animal Biotechnology, Bio-Resources Computing Research Center, Sustainable Agriculture Research Institute (SARI), Jeju National University, Jeju, Republic of Korea; ^7^Major of Aquaculture and Applied Life Sciences, Division of Fisheries Life Sciences, Pukyong National University, Busan, Republic of Korea

**Keywords:** beneficial bacteria, growth performance, innate immunity, gastrointestinal tract, challenge test, olive flounder

## Abstract

A basal diet without feed additives was used as a control (CON) and three diets were formulated by supplementing with *Bacillus subtilis* WB60 at 1 × 10^8^ CFU/g (Pro-A), *B. subtilis* SJ10 at 1 × 10^8^ CFU/g (Pro-B), *Enterococcus faecium* SH30 at 1 × 10^7^ CFU/g (Pro-C), and two other diets supplementing with antibiotics such as amoxicillin (AMO) at 4 g/kg and oxytetracycline (OTC) at 4 g/kg of the basal diet. A total of 450 fish averaging 12.1 ± 0.09 g (mean ± SD) were fed one of the six experimental diet groups in triplicates for 8 weeks. In disease resistance test, 45 fish from each group were intraperitoneally injected with the pathogenic bacteria, *Edwardsiella tarda*, and mortality was recorded for 15 days. At the end of 8-week feeding trial, weight gain, specific growth rate and feed efficiency of fish fed the Pro-A diet were significantly greater than those of fish fed the CON, OTC and AMO diets (*p* < 0.05). Furthermore, feeding efficiency and protein efficiency ratio of fish fed the Pro-A diet were significantly greater than those of fish fed the CON, OTC and AMO diets. Serum aspartate aminotransferase levels were significantly greater in fish fed the Pro-B diet than in those fed the Pro-A diet. The lysozyme activity of fish fed the Pro-A, Pro-B and Pro-C diets was significantly greater than that of the CON, OTC and AMO diets. The myeloperoxidase activity of fish fed the Pro-A diet was significantly greater than that of the fish fed the CON and AMO diets. The flounder growth hormone levels of fish fed the Pro-A, Pro-B, Pro-C and AMO diets were significantly greater than that of the fish fed the CON diet. The interleukin 1β gene expression levels in fish fed the Pro-B and Pro-C diets were significantly greater than those in fish fed the CON, OTC and AMO diets. The interleukin 10 gene expression levels in fish fed the Pro-A, Pro-B, Pro-C and OTC diets were significantly greater than those of fish fed the CON and AMO diets. Intestinal histology revealed that the average villi length of fish fed the Pro-A, Pro-B, and Pro-C diets were significantly greater than that of fish fed the CON, OTC and AMO diets. The cumulative survival rates of fish fed the Pro-A, Pro-B and Pro-C diets were significantly greater than those of fish fed the CON diet after the 15th day of the challenge test. Overall, the results demonstrated that the supplementation of fish-derived bacteria, *B. subtilis* (1 × 10^8^ CFU/g diet) or *E. faecium* (1 × 10^7^ CFU/g diet) in the diet could be the ideal probiotics to replace antibiotics in olive flounder fed FM reduced diet.

## Introduction

Olive flounder (*Paralichthys olivaceus* Temminck and Schlegel, 1846) is a flat fish native to the temperate coastal waters of East Asia that represents both an important capture as well as aquaculture industry. Olive flounder is most popular in Northeast Asian nations such as Korea, China, and Japan (1, 2). In South Korea, it is the most cultured species at more than 46,000 MT and accounts for more than 50% of the country’s overall production, which was 91,000 MT in 2022 (3). As olive flounder culture exceeds the capture fishery, there is an ever increasing pressure to reduce its reliance on fish meal (FM). FM accounts for up to 50% of the cost of olive flounder aqua feed. Although FM is an ideal source of essential amino acids (EAAs) and fatty acids (FAs), it comes at the expense of increased production for producers and great pressure on wild fisheries. Since the world’s production of FM is in decline and will unlikely increase in the future (4), the sustainability of the aquaculture industry is dependent on reducing reliance on this commodity moving forward. In olive flounder aquaculture, pathogenic bacteria *Edwardsiellatarda* infection (Edwardsiellosis) have become a major problem which cause severe economic losses. For this, farmers are mostly depend on the uses of antibiotics or vaccines for disease outbreak.

Fish meal replacement has been a major objective for aquaculture nutritionists for several decades. There are many candidates for FM replacement, such as meat and bone meal (5), poultry by-product meal (6), blood meal (7) and plant-based protein sources such as soybean meal (8). Proteins obtained from animal sources have great potential with some of the most popular sources being poultry byproduct meals, blood meals, and meat and bone meal (9). However, fish fed diets with low FM often suffer from issues related to growth, digestibility, palatability, and disease resistance. To overcome these problems, fish farmers have started to rely on various feed additives and antibiotics. The excessive application of the antibiotics is becoming a major health and environmental hazard since antibiotic-resistant strains are becoming increasingly prevalent (10, 11). Thus, the development of safe and sustainable alternatives to these pharmaceuticals has become an ever expanding field of research.

One of the most promising tools for reducing antibiotic reliance in the aquaculture industry is probiotics. Probiotics are microbes that help to correct imbalances in the microbiota of the intestines and confer benefits to the host organism’s health status when consumed in sufficient amounts (12). Some of the specific benefits of probiotics include their ability to retard and outcompete the growth of pathogens (13), aid in digestion by contributing to enzymatic activities (14), antiviral properties, enhancing immune responses (15, 16), reproductive performance (17), gut health and disease resistance (18) in fish. Recently, there have been tremendous interests in dietary intervention of host-associated or fish-derived probiotics research within the aquaculture industries (19–26). This kind of probiotic bacteria are generally isolated from fish intestine which would be more suitable in terms of enhanced growth and immune system in target animals than the so called probiotics using in aquaculture (13–18). In previous studies reported the positive single effect of the probiotics *Bacillus subtilis* WB60*, Bacillus subtilis* SJ10 and *Enterococcus faecium* in olive flounder. However, very few studies reported the efficacy of fish-derived multiple strains of probiotic bacteria in aquaculture. Therefore, in this study, we aimed to evaluate the comparison of dietary supplementation of the three fish-derivedprobiotics, *Bacillus subtilis* WB60, *Bacillus subtilis* SJ10 and *Enterococcus faecium* as well as two most widely used antibiotics in flounder aquaculture such as oxytetracycline and amoxicillin (27) based on the growth, immunity and disease resistance against *E. tarda* infection in juvenile olive flounder.

## Materials and methods

### Bacterial isolation and culture condition

The candidate probiotics, *B. subtilis* WB60 was isolated from the intestines of healthy Japanese eel (*Anguilla japonica*) and was identified by cluster analysis via 16S rDNA sequencing. The *B. subtilis* WB60 was isolated according to Lee et al. (28) and incubated at 30°C for 72 h in Luria-Bertani broth (LB broth; Sigma-Aldrich, St. Louis, USA), after which the optical density (OD600) was measured at 600 nm using spectrophotometry. The *B. subtilis* SJ10 was isolated from *jeotgal*, a traditional Korean fermented dish made from salt-preserved seafood such as squid, pollock roe, and shrimp, according to Hasan et al. (29) and it was incubated from a single colony on lysogeny broth (LB, USB Corporation, USA) agar, and was subsequently cultured in 10 mL of LB broth for 16 h at 37°C in a shaking incubator. Furthermore, *E. faecium* SH30 was isolated from the intestine of healthy Nile tilapia (*Oreochromis niloticus*), and the bacteria were grown in MRS (deMan, Rogosa, and Sharpe) broth at 36°C for 48 h according to Wang et al. (30). All probiotics were washed in sterile saline and the concentration of the final suspension was calculated to be 1 × 10^8^ CFU/g for WB60 and SJ10, and 1 × 10^7^ CFU/g for SH30 in the diets (28–30).

### Experimental fish and feeding trial

Juvenile olive flounder were obtained from a private farm (JUNGANG Fisheries, Chungcheongnam-do, Taean-gun, Republic of Korea). Prior to the start of the feeding trial, the apparent health status of the fish was checked visually, and the fish were starved for 24 h. All the fish were then fed a commercial diet for two weeks prior to the start of the feeding trial to acclimatize to the laboratory conditions. On average, 12.1 ± 0.09 g (mean ± SD) of fish were weighed, divided into triplicate groups of 25 fish corresponding to the dietary treatment, and randomly distributed into 18 indoor fiberglass tanks (40 L each) receiving a constant flow (1.2 L/min) of filtered seawater. During the experiment, supplemental aeration was provided in each tank to maintain adequate dissolved oxygen. The temperature was maintained at 19.0 ± 1.0°C throughout the experiment by electric heaters in a concrete reservoir. Fish were fed twice a day (09:00 and 19:00) for 8 weeks at a rate of 2.5 ~ 5% body weight per day. Dead fish were immediately removed and weighed, after which the amount of feed provided to the remaining fish was adjusted. The uneaten feed was siphoned 1 h after feeding. The inside of the tanks was scrubbed once per week to minimize algal and fungal growth.

### Experimental diets

The basal or control diet (**CON**) formulation is shown in Table 1. Anchovy fish meal (68.75% CP, crude protein) and soybean meal (47.04% CP) were used as the main protein sources, while fish oil was used as the main lipid source. The feed additives (probiotics) used in this experiment were **Pro-A**: *B. subtills* WB60 (1 × 10^8^ CFU/g) based on Lee et al. (28), **Pro-B**: *B. subtills* SJ10 (1 × 10^8^ CFU/g) based on Hasan et al. (29), **Pro-C**: *E. faecium* SH30 (1 × 10^7^ CFU/g) based on Wang et al. (30), as well as **OTC**: oxytetracycline, 4 g/kg diet and **AMO**: amoxicillin, 4 g/kg diet which were based on Won et al. (31). The procedures for feed manufacturing and preparation were performed as previously described by Lee et al. (28). According to the feed formulation table, all fine powdered ingredients were mixed thoroughly with an electric mixer (HYVM-1214, Hanyoung Food Machinery, Republic of Korea). Then, a stiff dough was formed by adding fish oil and the desired amount of water (~10%). The dough was passed through a pellet machine (SFD-GT, Shinsung, Republic of Korea) with a 0.2 cm die. The prepared diets were air-dried in a drying room for 48 h, broken into smaller pieces and stored at -20°C. According to the proximate composition analysis, shown in Table 2, all the diets were iso-nitrogenous and iso-lipidic.

**Table 1:** Composition of the basal diet (% of dry matter basis).

Ingredients	%
Anchovy fish meal^1^	45
Soybean meal	12
Starch^2^	3.8
Wheat flour	7.0
Blood meal	4.5
Squid liver powder	5.6
Meat and bone meal	8.0
Poultry by product meal	4.5
Fish oil^3^	4.3
Vitamin premix^4^	1.2
Mineral premix^5^	1.2
etc.^6^	3.0
Proximate analysis (% of DM basis)	
Moisture	8.56
Crude protein	56.2
Crude lipid	8.35
Crude ash	11.4

^1Suhyup Feed Co. Uiryeong, Korea.^

^2The Feed Co. Goyang, Korea.^

^3Jeil Feed Co. Hamman, Korea.^

^4^Contains (as mg/kg in diets): Ascorbic acid, 300; dl-Calcium pantothenate, 150; Choline bitate, 3,000; Inositol, 150; Menadion, 6; Niacin, 150; Pyridoxine HCl, 15; Rivoflavin, 30; Thiamine mononitrate, 15; dl-α-Tocopherol acetate, 201; Retinyl acetate, 6; Biotin, 1.5; Folic acid, 5.4; Cobalamin, 0.06.

^5^Contains (as mg/kg in diets): NaCl, 437.4; MgSO_4_·7H_2_O, 1379.8; ZnSO_4_·7H_2_O, 226.4; Fe-Citrate, 299; MnSO_4_, 0.016; FeSO_4_, 0.0378; CuSO_4_, 0.00033; Ca(IO)_3_, 0.0006; MgO, 0.00135; NaSeO_3_, 0.00025.

^6etc.: Calcium phosphate; Lecithin, Batain; Taurine; Choline; Vitamin C; Lysine; Methionine.^

**Table 2:** Proximate composition of the eight experimental diets (% of DM basis).^1^

Parameters	Diets					
	CON	Pro-A	Pro-B	Pro-C	OTC	AMO
Moisture	8.56	9.01	8.91	8.56	8.91	8.71
Crude protein	56.2	55.9	54.8	57.1	55.9	56.1
Crude lipid	8.35	8.29	8.34	8.23	8.11	8.32
Crude ash	11.4	10.9	11.2	10.9	11.2	11.8

^1^Values are means of triplicate samples. Values in each row without superscripts are non-significantly different (*P* > 0.05).

### Sample collection and analysis

At the end of the feeding trial, fish were starved for 24 h prior to sample and data collection. The fish were subsequently counted and weighed to calculate the final weight (FW), weight gain (WG), specific growth rate (SGR), feed efficiency (FE) protein efficiency ratio (PER) and survival (SR). Four fish from each tank were selected at random, weighed individually, and dissected to obtain liver and visceral metrics for calculation of hepatosomatic index (HSI) and visceral somatic index (VSI); thereafter, the same intestinal samples were used for histological observation and enzyme activity. Three additional fish per tank were captured at random and anesthetized with ethylene glycol phenyl ether (200 mg/L for 5–10 min). After this, blood was drawn from the caudal vein, which was subsequently centrifuged at 5000 × g for 10 min to obtain the serum. Serum samples were then stored at −70°C for the analysis of non-specific immune responses, such as lysozyme, and myeloperoxidase (MPO) activities, in addition to biochemical parameters, including aspartate aminotransferase (AST), alanine aminotransferase (ALT), glucose and total protein (TP) levels. The serum levels of AST, ALT, glucose, and total protein were determined by a chemical analyzer (Fuji DRI-CHEM 3500i, Fuji Photo Film Ltd., Tokyo, Japan) following the manufacturer’s instructions.

Three additional fish from each tank were collected for whole-body proximate composition analysis. Proximate composition analyses of both whole fish and experimental diets were performed by the standard methods of AOAC (32). Whole fish and diet samples were dried at 105°C to a constant weight to determine their moisture content. The ash content was determined by incinerating the samples at 550°C. The protein concentration was determined by using the Kjeldahl method (N × 6.25) after acid digestion. Crude lipids were measured by Soxhlet extraction using Soxhlet system 1,046 (Tacator AB, Hoganas, Sweden) after the samples were freeze-dried for 20 h.

### Antioxidant capacity and non-specific immune response analyses

Olive flounder serum lysozyme activity was analyzed as follows: 0.1 mL of test serum was added to 2 mL of a suspension of *Micrococcus lysodeikticus* (0.2 mg/mL) in 0.05 M sodium phosphate buffer (pH 6.2). The reactions were carried out at 20°C, and the absorbance was measured at 530 nm. Measurements were taken between 0.5 min and 4.5 min on a spectrophotometer. One lysozyme activity unit was defined as the amount of enzyme that produced a decrease in absorbance corresponding to 0.001/min. Myeloperoxidase activity was measured according to the method described by Quade and Roth (33). Briefly, 20 μL of serum was diluted with Hank’s balanced salt solution (HBSS) without Ca^2+^ or Mg^2+^ (Sigma- Aldrich) in 96-well plates. Then, 35 μL of 3, 3′, 5, 5′ tetramethylbenzidine hydrochloride (TMB, 20 mM) (Sigma-Aldrich) and H_2_O_2_ (5 mM) were added. The color change reaction was stopped after 2 min by adding 35 μL of 4 M sulfuric acid. Finally, the optical density was read at 450 nm in a microplate reader.

### Real-time PCR

Tissue fragments from intestine of fish were obtained and immediately stored at −80°C in TRIzol reagent (Thermo Fisher Scientific) for RNA extraction. Total RNA was extracted from 0.5 g of olive flounder tissue using TRIzol Reagent (Thermo Fisher Scientific, San Jose, CA, USA). Afterwards, the RNA was quantified and the purity was assessed spectrophotometrically. The RNA was then treated with DNase I (Cosmogenetech, Seoul, Republic of Korea) to remove genomic DNA contamination. Complementary DNA (cDNA) was synthesized using M-MuLV reverse transcriptase (Cosmogenetech). The expressions of three selected immune-related genes were analyzed by real-time quantitative polymerase chain reaction (RT-qPCR), which was performed with a Bio-Rad CFX96 (Bio-Rad, Hercules, CA, USA) using SYBR Green PCR Core Reagents (Cosmogenetech). The relative expression levels of the target gene transcripts such as flounder growth hormone (FGH), interleukin-1beta (IL-1*β*) and interleukin-10 (IL-10) were measured with β-actin as an internal control were using CFX Manager software version 2.0 (Bio-Rad) (Table 3). In all the cases, each PCR was performed with triplicate samples.

**Table 3:** Gene specific primers, amplicon lengths and gene bank accession numbers of immune and growth-related genes used in this study.

Name of gene	Sense	Oligonucleotide sequence (5′ to 3′)	Base pair (bp)	Gene bank accession number
β-actin	F	CAGCATCATGAAGTGTGACGTG	107	HQ386788.1
	R	CTTCTGCATACGGTCAGCAATG		
FGH	F	CGCCGTATGGAAACTCTGAACT	160	M23439.1
	R	GGGTGCAGTTAGCTTCTGGAAA		
IL-1β	F	ATGGAATCCAAGATGGAATGC	250	AB720983.1
	R	GAGACGAGCTTCTCTCACAC		
IL-10	F	AGCGAACGATGACCTAGACACG	114	KF025662.1
	R	ACCGTGCTCAGGTAGAAGTCCA		

^1FGH, flounder growth hormone.^

^2IL-1β, interleukin-1beta.^

^3IL-10, interleukin-10.^

### Histology

The anterior intestinal tissues from the fish (n = 3) were dissected and fixed in 10% neutral buffered formalin, dehydrated in a graded ethanol series and embedded in paraffin. The tissue blocks were sectioned by a microtome machine (HistoCore AUTOCUT, Leica Biosystems, Germany) each of 4 μm thick and stained with hematoxylin and eosin (H&E). At least 6 tissue sections from each sample were examined for intestinal villi length (small fingerlike projections protruded from the intestinal wall) under a light microscope (AX70 Olympus, Tokyo, Japan) equipped with a digital camera (DIXI Optics, Daejeon, Republic of Korea), and an image analysis software (Image J 1.32j, National Institute of Health, USA). Data are presented as means ± SE.

### Challenge test

After sampling, seven fish from each tank were redistributed into 18 tanks in a non-recirculating system without water renewal to perform the 15 days of challenge test. The pathogenic bacterium, *Edwardsiella tarda* (*E. tarda*) FSW910410 was obtained from the Department of Biotechnology, Pukyong National University, Busan, Rep. Korea. The bacteria were originally sourced from diseased olive flounder and cultured on tryptic soy agar (TSA, Sigma) plates (24 h at 27°C). All the fish were subjected to intraperitoneal injection of 50 μL of active *E. tarda* (3 × 10^8^ CFU/mL) solution. The water temperature was maintained at 19 ± 1.0°C (mean ± SD) during 15 days of challenge test and fish mortalities were recorded daily from each tank. Dead fish were necropsied and kidney samples were taken and streaked on Salmonella-Shigella agar (SS agar, Difco). The presence of black pigments confirmed *E. tarda* infection.

### Statistical analysis

The results are presented as means ± SE (number of replicates as indicated). All the data were analyzed by one-way ANOVA (Statistix 3.1; Analytical Software, St. Paul, MN, USA) to test the effects of the dietary treatments. Prior to the one-way ANOVA, normality and homogeneity of variances were checked. When a significant treatment effect was observed, an LSD *post hoc* test was used to compare the means. Treatment effects were considered significant at the *p* < 0.05 level.

## Results

### Growth performance and feed utilization in fish

Table 4 shows the growth performance and feed utilization of juvenile olive flounder fed different experimental diets for 8 weeks. At the end of the feeding trial, WG and SGR of fish fed the Pro-A diet were significantly higher greater those of fish fed the CON diet (*p* > 0.05). The FE of fish fed the Pro-B diet was significantly greater than that of fish fed the CON, OTC, or AMO diets (*p* > 0.05). The PER of fish fed the Pro-A diet was significantly greater than that of fish fed the CON and AMO diets (*p* > 0.05). Moreover, probiotics supplemented diets were not significantly different from fish fed the Pro-A, Pro-B and Pro-C diets (*p* > 0.05) in terms of growth performance. Furthermore, there were no significant differences (*p* > 0.05) in terms of SR, CF, HSI or VSI among fish fed the experimental diets.

**Table 4:** Growth performance and feed utilization of olive flounder fed the six experimental diets for 8 weeks.^1^

Parameters	Diets						
	CON	Pro-A	Pro-B	Pro-C	OTC	AMO	*p*-value
IBW^2^	12.5 ± 0.4	12.4 ± 0.3	12.2 ± 0.2	12.4 ± 0.3	12.4 ± 0.3	12.4 ± 0.3	0.36
FBW^3^	36.7 ± 4.2^b^	42.0 ± 3.2^a^	40.8 ± 4.5^ab^	40.7 ± 3.4^ab^	36.9 ± 4.5^b^	37.0 ± 1.4^b^	0.04
WG (%)^4^	194 ± 2.3^b^	230 ± 10.1^a^	224 ± 8.3^ab^	229 ± 8.3^ab^	202 ± 4.0^b^	199 ± 2.3^b^	0.03
SGR (%/day)^5^	1.95 ± 0.2^b^	2.24 ± 0.2^a^	2.16 ± 0.2^ab^	2.16 ± 0.2^ab^	2.01 ± 0.1^b^	1.99 ± 0.1^b^	0.03
FE (%)^6^	106 ± 7.8^b^	118 ± 4.5^ab^	125 ± 5.5^a^	115 ± 7.2^ab^	111 ± 7.8^b^	110 ± 2.3^b^	0.03
PER^7^	0.65 ± 0.1^b^	0.84 ± 0.1^a^	0.76 ± 0.1^ab^	0.76 ± 0.1^ab^	0.70 ± 0.1^ab^	0.66 ± 0.1^b^	0.04
Survival (%)^8^	93.3 ± 0.2	90.7 ± 0.2	89.3 ± 0.2	89.3 ± 0.2	96.0 ± 0.1	98.7 ± 0.1	0.12
HSI (%)^9^	1.24 ± 0.1	1.66 ± 0.3	1.29 ± 0.4	1.31 ± 0.3	1.13 ± 0.2	1.12 ± 0.4	0.22
VSI (%)^10^	2.05 ± 0.5	1.94 ± 0.2	1.74 ± 0.7	1.94 ± 0.1	1.88 ± 0.6	1.74 ± 0.2	0.12
CF (%)^11^	0.92 ± 0.1	0.97 ± 0.2	0.93 ± 0.1	0.93 ± 0.2	0.92 ± 0.1	0.91 ± 0.1	0.32

^1^Values are means from triplicate groups of fish where the values in each row with different superscripts (a,b) are significantly different (*p* < 0.05). Data are presented as means ± SE.

^2IBW, initial body weight.^

^3FBW, final body weight.^

^4^WG, Weight gain (%) = (final weight - initial weight) / initial weight × 100.

^5^SGR, specific growth rates (%/day) = (log_e_ final weight - log_e_ initial weight) / days × 100.

^6^FE, feed efficiency (%) = (body weight gain / dry feed intake) × 100.

^7^PER, protein efficiency ratio = (final weight - initial weight) / protein intake.

^8^SR (%) = (total fish – dead fish) / total fish × 100.

^9^HSI, hepatosomatic index (%) = liver weight / body weight × 100.

^10^VSI, viscerosomatic index (%) = viscerai weight / body weight × 100.

^11^CF, condition factor (%) = body weight / body length^3^ × 100.

### Whole-body proximate composition

There were no significant differences (*p* > 0.05) in terms of crude protein, lipid, moisture or ash content among any of the group of fish fed the experimental diets for 8 weeks (Table 5).

**Table 5:** Whole-body proximate composition of olive flounder fed the experimental diets.^1^

Parameters	Diets						
	CON	Pro-A	Pro-B	Pro-C	OTC	AMO	*P*-value
Moisture	76.3 ± 1.2	75.5 ± 2.6	75.1 ± 0.7	75.8 ± 1.6	76.8 ± 0.5	76.2 ± 1.2	0.22
Protein	20.3 ± 0.1	21.0 ± 0.2	20.8 ± 0.2	21.5 ± 0.3	22.3 ± 0.1	20.7 ± 0.3	0.32
Lipid	2.42 ± 0.1	2.39 ± 0.1	2.30 ± 0.1	2.34 ± 0.1	2.41 ± 0.1	2.41 ± 0.1	0.42
Ash	4.09 ± 0.2	4.25 ± 0.3	4.21 ± 0.3	4.16 ± 0.4	4.14 ± 0.1	4.19 ± 0.3	0.31

^1^Values are means from triplicate groups of fish (*n* = 3) where the values in each row without superscripts are non-significantly different (*p* > 0.05). Data are presented as means ± SE.

### Hematological parameters

**Figure 1:**
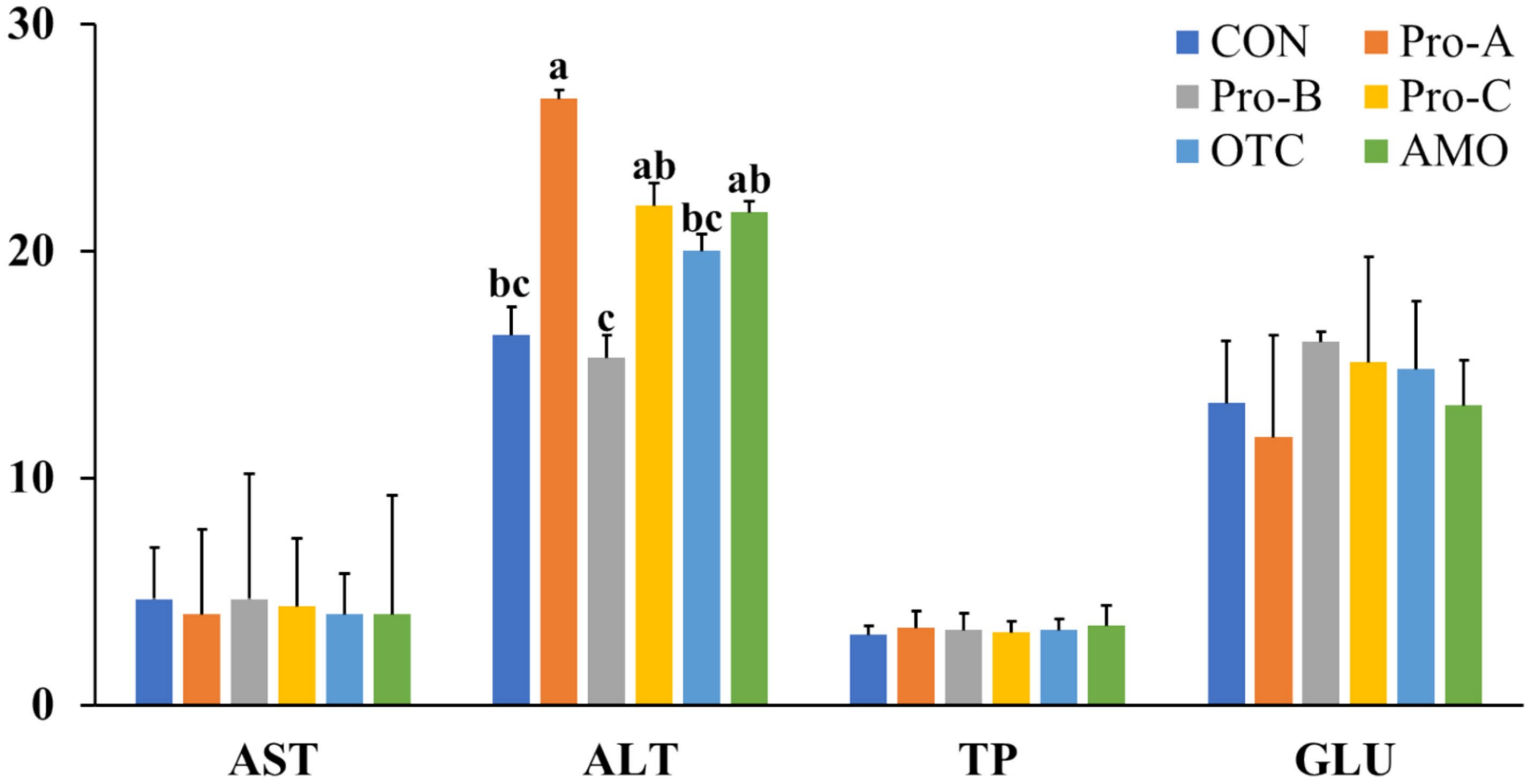
Hematological parameters of olive flounder fed the six experimental diets. Values are means from triplicate groups of fish (*n* = 3) where the values in each row with different superscripts (a,b,c) are significantly different (*p <* 0.05). AST: Aspartate aminotransferase (U/l), ALT, alanine aminotransferase (U/l), GLU, glucose (mg/dl), TP: Total protein (g/dl). Data are presented as means ± SE.

The blood parameters of the juvenile olive flounder fed the experimental diets are shown in Figure 1. The serum ALT level in the fish fed the Pro-A diet was significantly lower than those of fish fed the Pro-A, Pro-C and AMO diets (*p* < 0.05). There were no significant differences in AST, glucose, or total protein content among any of the group of fish fed the experimental diets for 8 weeks (*p* > 0.05).

### Non-specific immune responses

**Figure 2:**
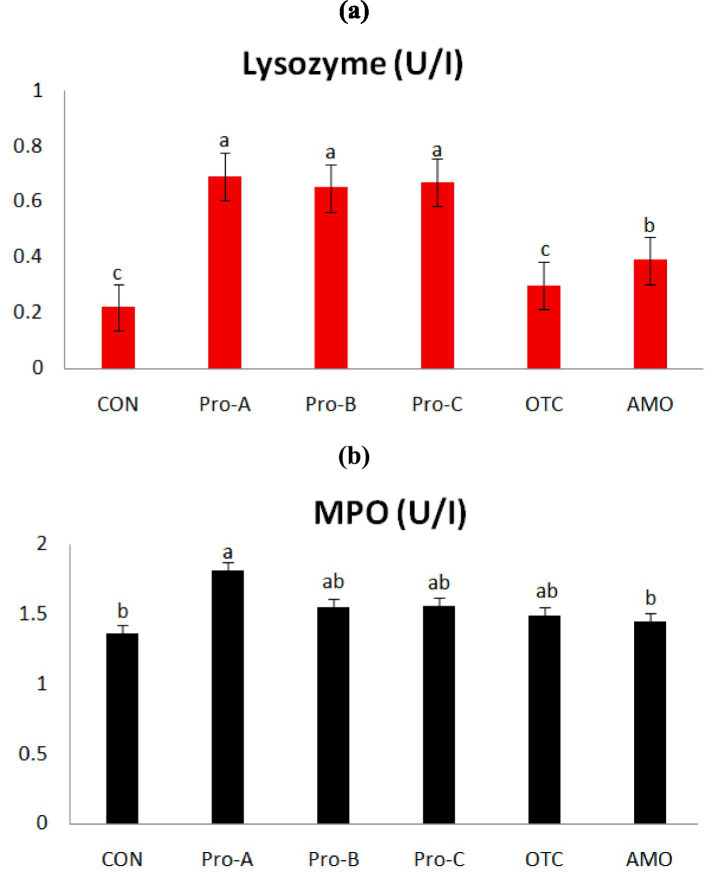
Non-specific immune responses of olive flounder fed the six experimental diets. Values are means from triplicate groups of fish (*n* = 3) where the values in each row with different superscripts (a,b,c) are significantly different (*p* < 0.05); **(A)** Lysozyme (U/ml): lysozyme activity and **(B)** MPO (absorbance): myeloperoxidase (OD at 450 nm). Data are presented as means ± SE.

Figure 2 shows the non-specific immune responses of juvenile olive flounder fed different experimental diets for 8 weeks. The serum lysozyme activities of fish fed the Pro-A, Pro-B and Pro-C diets were significantly greater than that of fish fed CON,OTC and AMO diets (*p* > 0.05). Myeloperoxidase activity of fish fed Pro-A diet was significantly higher than those from the CON and AMO diets (*p* > 0.05). However, there were no significant differences in MPO activities among fish fed the CON, Pro-B, Pro-C, OTC and AMO diets.

### Growth and immune related gene expressions

**Figure 3:**
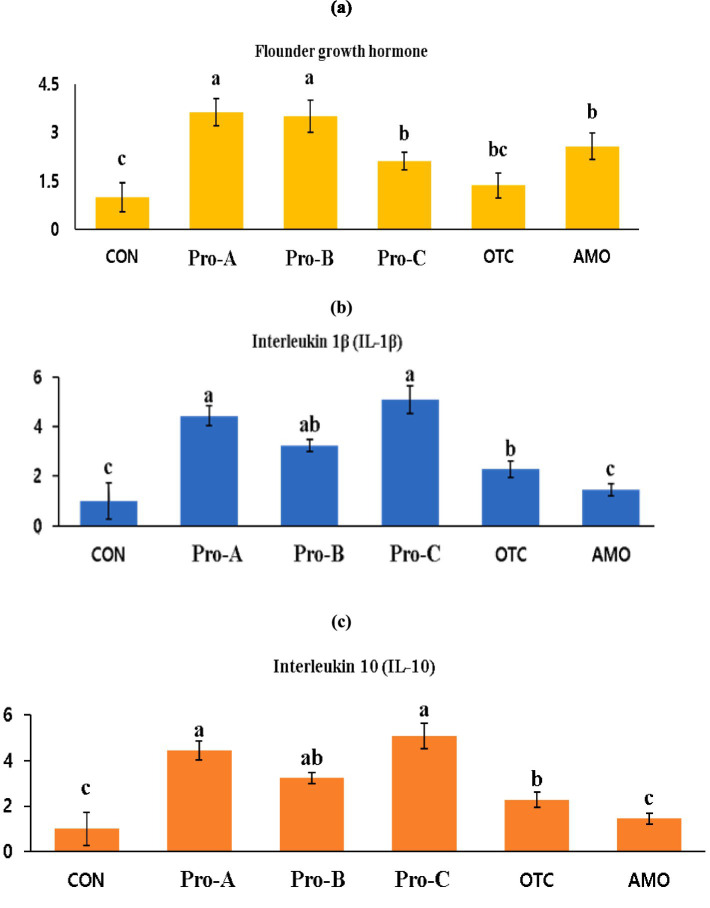
Relative mRNA gene expression levels of **(A)** flounder growth hormone (FGH), **(B)** interleukin-1 beta (IL-1β) and **(C)** interleukin-10 (IL-10) of intestine from olive flounder fed the experimental diets for 8 weeks. Data are presented as means ± SE.

The gene expression profiles of the immunological parameters in the intestine of olive flounder fed diets supplemented with probiotics are presented in Figure 3. The mRNA expression levels of the FGH in fish fed the Pro-A and Pro-B diets were significantly greater than those in fish fed the CON, Pro-C, OTC and AMO diets (*P* < 0.05). However, there were no significant differences in FGH concentrations in fish fed the CON and OTC diets (*p* > 0.05). The IL-1β expressions in fish fed the Pro-A, Pro-B and Pro-C diets were significantly greater than those in fish fed the CON and AMO diets (*P* < 0.05). The IL-10 expressions in fish fed the Pro-A and Pro-C diets were significantly greater than those in fish fed the CON, OTC and AMO diets (*P* < 0.05). However, there were no significant differences in IL-10 mRNA expressions in fish fed the CON and AMO diets (*p* > 0.05).

### Histology

**Figure 4:**
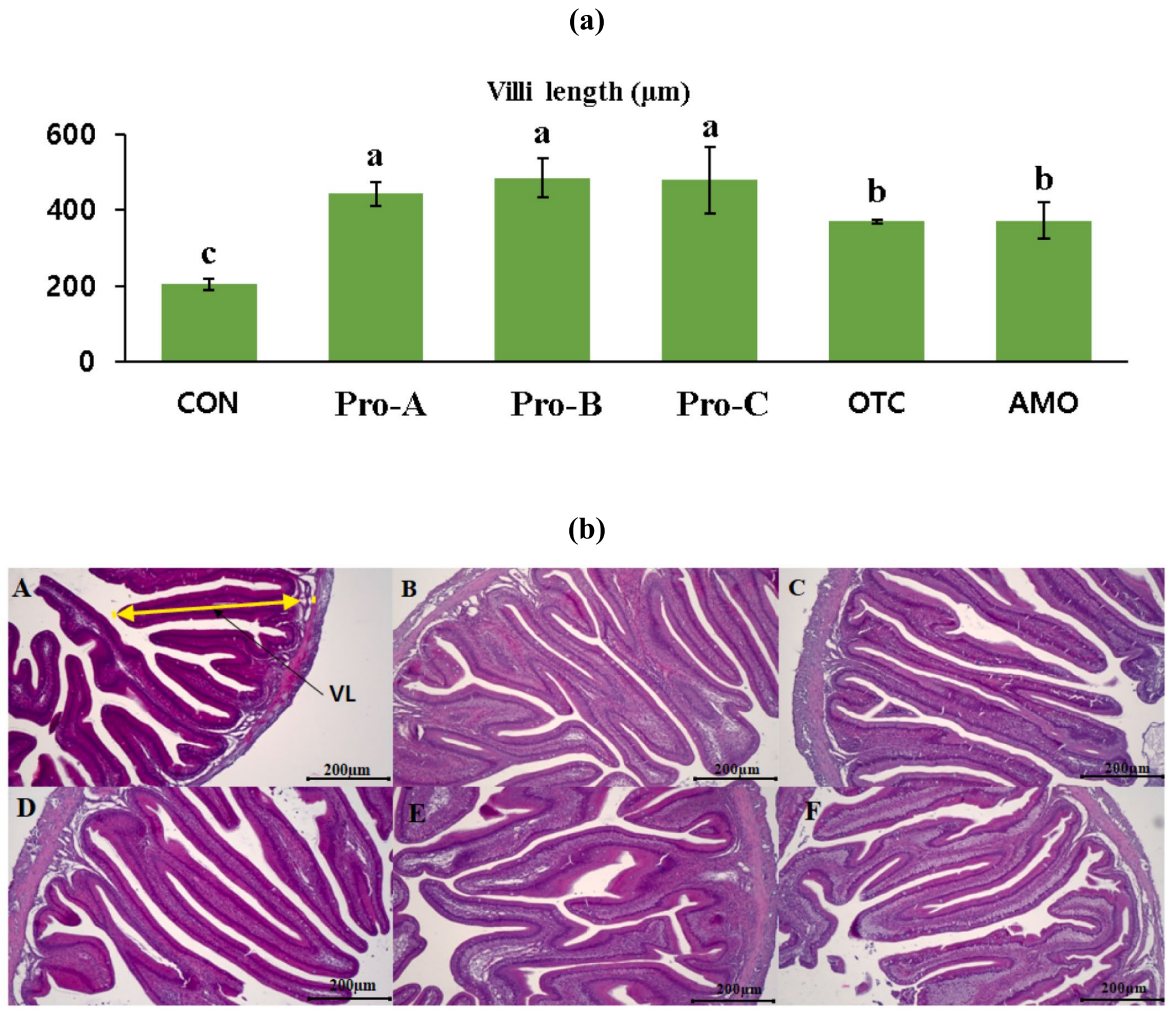
Intestinal histology of juvenile olive flounder fed the experimental diets for 8 weeks; **(a)** villi lengths in fish fed the different diets; **(b)** histological photomicrographs of **(A)** CON **(B)** Pro-A **(C)** Pro-B **(D)** Pro-C **(E)** OTC **(F)** AMO diet groups; (scale bar = 200 μm; original magnification×40).

Histological analysis of the anterior intestine of olive flounder fed different experimental diets for 8 weeks is shown in Figure 4. The intestinal villi length in fish fed the Pro-A, Pro-B and Pro-C diets had significantly greater villi lengths than the fish fed the CON, OTC and AMO diets (Figure 4A). The fish fed the Pro-A, Pro-B, Pro-C, OTC and AMO diets clearly exhibited better intestinal histomorphology with more massive villi in comparison to the CON diet (Figure 4B). In addition, the images which corresponds to the CON group, shows certain irregular shape and improper arrangement of villi in fish fed the CON, OTC and AMO diets compared to those of the Pro-A, Pro-B and Pro-C groups.

### Challenge test

**Figure 5:**
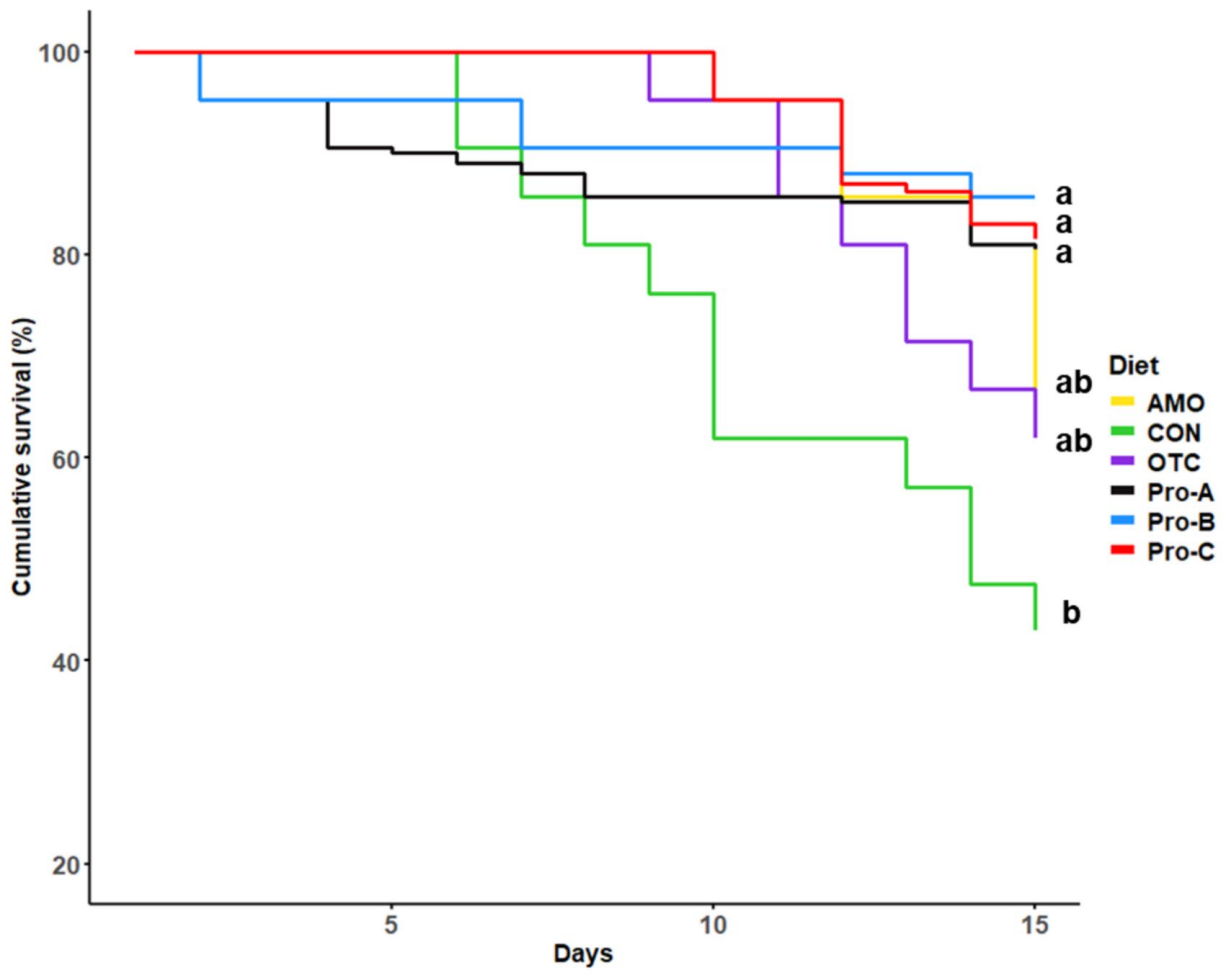
Cumulative survival (%) of olive flounder during 15 days challenge test. For the survival analysis, we used R 4.4.1 (R Development Core Team, 2023) with the survival and survminer packages to perform Kaplan–Meier survival analysis. This approach allowed us to estimate survival curves and compare survival rates among groups using the log-rank test. Specifically, the Kaplan–Meier method (via the survfit function) was employed to analyze survival data across different dietary treatments.

The percent cumulative survival of juvenile olive flounder challenged with *E. tarda* for 15 days is shown in Figure 5. During the challenge test, the first mortalities occurred on the second day. At the end of the 15 day challenge, the percent cumulative survivals of fish fed the Pro-A, Pro-B, and Pro-C diets were significantly greater than that of fish fed the CON diet (*p <* 0.05). However, there were no significant differences in cumulative survival rates in fish fed the Pro-A, Pro-B, Pro-C, OTC, and AMO diets (*p >* 0.05).

## Discussion

Research into the use of probiotics in aquaculture nutrition has attracted much interest due to their health benefits and because they are considered environmentally friendly (34). In recent years, probiotic effects have been studied in different fish species (35). Additionally, many trials have investigated the extraction of probiotic strains from the intestines of various fish, which are subsequently added to aquafeeds. This strategy of sourcing and using probiotics from these species has improved growth performance, feed efficiency and immune response (36, 37). Therefore, to build on this growing body of knowledge, the present study utilized three different probiotics according to the results of previous experiments.

The two probiotics used were *Bacillus subtilis* extracted from the intestines of Japanese eel and *jeotgal* (28, 29), because most other probiotic studies have focused on the use of *Bacillus* spp. (34–41). The other probiotics used was *E. faecium* isolated from the intestine of healthy Nile tilapia (18). In this study, we used dietary live or active probiotics during feeding of fish to ensure direct interaction with fish physiology in terms of gut microbiome and mucosal immunity. Moreover, a previous experiment in which Pirarucu, *Arapaima gigas* was fed diets containing live probiotics, *E. faecium* 1 × 10^8^ CFU/g showed increased weight gain compared to that in the control treatment group (42). The results of the present study showed that all the experimental diets containing the probiotics were equally effective and resulted in increased weight gain and feed efficiency compared with the control diet. These results are likely due to the increased secretion of proteolytic enzymes, which increase feed efficiency, similar to the findings of probiotic experiments in olive flounder (35). Likewise, beneficial effects of probiotics have also been reported on growth performance in terms of improving weight gain, specific growth rate, and feed efficiency in tilapia (18, 30, 43, 44), whiteleg shrimp (31), Japanese eel (28, 36), starry flounder (39), red sea bream (26), rainbow trout (16, 37, 40), Pirarucu (42) and olive flounder (29, 35). In agreement of the present study, Won et al. (31, 41) reported that *B. subtilis* WB60 at 1 × 10^8^ CFU/g can enhance growth and feed utilization in whiteleg shrimp and Nile tilapia. Furthermore, probiotic bacteria, *E. faecium* at 1 × 10^7^ CFU/mL in water showed significantly better final weight and daily weight gain in tilapia which endorsed the results of the present study (30).

Modulation of the immune system is one of the most common benefits of probiotics (44). Lysozyme activity is frequently used as an indicator of non-specific immune functions and is the principle means of responding infections in fish (28). This enzyme not only has bacteriolytic activity against gram-positive and gram-negative bacteria (45), but also has anti-inflammatory and antiviral properties. The MPO is another important enzyme that utilizes oxidative radicals to produce hypochlorous acid, which kills pathogens (29). In the present study, the immune parameters, lysozyme including MPO activities were measured and the beneficial effects of both probiotic bacteria on nonspecific immune related enzyme responses, were clearly shown to be greatest for the olive flounder fed *B. subtilis*, at the 1 × 10^8^ CFU/g (Pro-A and Pro-B) and *E. faecium*, at the 1 × 10^7^ CFU/g (Pro-C). In agreement of the present study, previous research findings reported that dietary supplementation of BSWB60, BSSJ10 and EFSH30 in the diets can improve the lysozyme and MPO activities in olive flounder (28–30).

Growth hormone is a hormone that stimulates the secretion of IGF-1 in the liver, increases the concentration of glucose and vitreous acid (46), produces IGF-1 induced protein synthesis (47), and is reported to be an indicator of growth factors in fish such as promoting cell division (48). In the present study, olive flounder fed probiotics supplemented diets exhibited significantly greater FGH expression than did those fed the control diet (Figure 3A). Similarly, previous studies in which probiotics were added showed high FGH value (49, 50). These studies have reported that probiotic supplementation can affect growth hormone-related gene expression in fish (28, 29). In consistent of the present study, Jang et al. (26) found that dietary supplementation of *Bacillus* sp. as a host-associated probiotic in red sea bream can enhance the growth hormone in fish which is also reflected in the growth performance of fish.

The IL-1β is one of the earliest expressed pro-inflammatory cytokines and enables organisms to respond promptly to infection by inducing a cascade of reactions leading to inflammation (51). Many of the effector roles of IL-1β are mediated through the up- or down-regulation of the expressions of other cytokines and chemokines (52). The IL-1β was the first interleukin to be characterized in fish and has since been identified in a number of fish species, such as rainbow trout (53), carp (54), seabass (55), gilt head seabream (56), haddock (57), tilapia (58). The IL-10 on the other hand, is an anti-inflammatory cytokine that down-regulates the expression of pro-inflammatory cytokines (59). Additionally, IL-10 was initially discovered to be an inhibitory factor for the production of Th1 cytokines. Subsequently, pleiotropic inhibitory and stimulatory effects of IL-10 on various types of blood cells were described, including its role as a survival and differentiation factor for B cells. The IL-10, which is produced by activated monocytes, T cells and other cell types, such as keratinocytes, appears to be a crucial factor for at least some forms of peripheral tolerance and a major suppressor of the immune response and inflammation. The inhibitory function of IL-10 is mediated by the induction of regulatory T cells (60). In the present study, the activities of IL-1β and IL-10 in the intestine of the fish that were administered probiotics were significantly greater than those in the control with low FM diet. Therefore, dietary probiotics appears to increase the immune function of fish even though fish were offered with low FM diets. The results suggest that dietary supplementation of probiotics might be attenuated the inflammation in fish through up-regulating the cytokine gene (IL-1β and IL-10) expressions in fish. In agreement of the present study, Lara-Flores et al. (43) and Back et al. (50) reported that probiotics supplemented in low protein or low FM diets can enhance the immunity in tilapia and olive flounder, respectively without compromising the health status of fishes.

Intestinal morphological parameters (villus length and muscular layer thickness) are indicative of a healthy gut in fish. The intestine is very important for the digestion and absorption of nutrients. The length of the intestinal villi determines the absorption of nutrients in the GI tract (gastrointestinal tract) (61, 62). Thus, digestive function is associated with intestinal development (62). In this study, the beneficial effects of probiotics on intestinal morphology were clearly observed. The length of the villi increased in a dose dependent manner and villi length was significantly highest for the olive flounder fed the Pro-A, Pro-B, and Pro-C diets (Figure 4A). In the same manner, Lee et al. (28) reported that probiotics are capable of increasing the villus length in the proximal intestine of Japanese eel. Furthermore, Won et al. (31, 41) postulated that dietary *Bacillus subtilis* WB60 can increase the villus length and muscular thickness in the intestine of whiteleg shrimp and Nile tilapia which supported the results of the present study.

With regard to disease resistance, olive flounder fed with probiotics, Pro-A and Pro-B at 1 × 10^8^ CFU/g as well as Pro-C at 1 × 10^7^ CFU/g supplemented in the diet exhibited the highest disease resistance compared to the control group. However, there were no significant differences in cumulative survival rates in fish fed the Pro-A, Pro-B, Pro-C, OTC and AMO diets suggesting that the three fish-derived probiotics were equally effective to the antibiotics. In this study, the mode of probiotic action is attributed to follow the host-specific (63) and strain-specific (64) properties. The major factors that affecting the disease resistance in fish perhaps the origin, source, viability and dose of the probiotics and their duration of administration (65–68). Likewise, in recent studies, it is reported that dietary supplementation of host-associated probiotic bacteria *B. subtilis* and *E. faecium* could enhance the disease or stress resistance in Japanese eel, shrimp, hybrid yellow catfish, Chinese perch, hybrid grouper, olive flounder, rainbow trout, red sea bream and tilapia (20–31, 41). In consistent with the present study, it has been reported that the higher levels of lysozyme improved disease resistance in infected fish such as Atlantic salmon challenged with *Aeromonas salmonicida* (69) and sheatfish challenged with *Edwardsiella tarda* (70).

## Conclusion

In conclusion, the present study revealed the potential benefits of supplementation with the bacteria species, *B. subtilis* and *E. faecium* as probiotics in the diet of olive flounder. Therefore, *B. subtilis* at 1 × 10^8^ CFU/g and *E. faecium* at 1 × 10^7^ CFU/g could be ideal probiotics for improving growth performance, immune responses, enzyme activity and disease resistance, while replacing the dietary supplementation of antibiotics in juvenile olive flounder fed a reduced FM diet. The utilization of these probiotics could help to further enhance the olive flounder production in the farm level and to replace indiscriminate use of antibiotics without compromising health status in fish as well as to reduce environmental pollution in terms of antimicrobial resistance with higher consumer acceptance. However, further research is warranted to evaluate the effects of the current probiotics on the diversity of intestinal microbiota and immunity on gut-brain axis in fish.

## Data Availability

The datasets presented in this study can be found in online repositories. The names of the repository/repositories and accession number(s) can be found at: https://www.ncbi.nlm.nih.gov/genbank/, HQ386788.1, https://www.ncbi.nlm.nih.gov/genbank/, M23439.1, https://www.ncbi.nlm.nih.gov/genbank/, AB720983.1, https://www.ncbi.nlm.nih.gov/genbank/, KF025662.1.
